# Exploring the do-it-yourself approach in subjective refraction

**DOI:** 10.1371/journal.pone.0334644

**Published:** 2025-10-16

**Authors:** Raquel Salvador-Roger, José J. Esteve-Taboada, Sara Ferrer-Altabás, Vicente Micó, Alberto Domínguez-Vicent, Abinaya Priya Venkataraman

**Affiliations:** 1 Department of Optics and Optometry and Vision Sciences, University of Valencia, Burjassot, Spain; 2 Division of Eye and Vision, Department of Clinical Neuroscience, Karolinska Institute, Stockholm, Sweden; LV Prasad Eye Institute, INDIA

## Abstract

**Purpose:**

To evaluate the precision and accuracy of a novel do-it-yourself (DIY) subjective refraction technique utilizing a manual tunable spherical lens and an adjustable astigmatic lens.

**Methods:**

Sixty-six participants performed three self-refraction measurements using a DIY approach, while an optometrist conducted one measurement using the traditional subjective refraction methodology. The DIY approach relied on power vector analysis, where a tunable spherical lens and an adjustable astigmatic lens (Stokes lens), aligned to the participant’s eye, were rotated to obtain measurements. The study assessed refractive error, visual acuity, and required measurement time. Repeatability was evaluated using the coefficient of repeatability, and agreement between methods was analyzed using limits of agreement.

**Results:**

The coefficients of repeatability for refractive error components (M, J_0_, J_45_) were ±0.38 D, ± 0.21 D, and ±0.21 D, respectively. Visual acuity showed a repeatability of ±0.06 logMAR, and the required time had a repeatability of ±55 seconds. The limits of agreement between the DIY approach and the traditional method were ±0.64 D, ± 0.36 D, and ±0.26 D for refractive error components, ± 0.04 logMAR for visual acuity, and ±127 seconds for required time.

**Conclusions:**

The DIY approach can be considered a repeatable alternative among self-subjective refraction methods, although it is a first attempt and not yet fully automatic. While it cannot supplant traditional subjective refraction and further research would be required to address, it has the potential to be a viable option in certain specific cases, such as circumstances characterized by a lack of eye care professionals or humanitarian works.

## Introduction

The accurate determination of refractive errors is a cornerstone of clinical optometry and ophthalmology [1]. Subjective refraction remains the gold standard for assessing and prescribing corrective lenses, as it allows for individualized optimization of visual acuity (VA) [2]. Despite advances in objective refraction methods, the subjective component is indispensable for fine-tuning the final prescription and accounting for the patient’s perceptual preferences [2].

Over the years, several methods and tools have been developed to enhance the accuracy and efficiency of subjective refraction [3–6] with more recent advancements including approaches based on the vector representation of refractive errors, such as power vectors [5,7,8]. Despite all these attempts, conventional phoropters, combined with techniques like the Jackson Cross-cylinder and the red-green duochrome test, are still the standard in clinical practice.

Parallel to these advancements, novel optical technologies such as tunable liquid lenses (TLLs) have emerged. These TLLs, with their adjustable focal power, provide unprecedented flexibility for real-time spherical or astigmatic adjustments by manually rotating an external ring or by varying the electronically applied current [9–13]. TLLs can offer the same range of dioptric power as a conventional phoropter, but in a continuous manner [14], while also presenting a more compact and lightweight design [9,10]. On the other hand, adjustable astigmatic devices as the Stokes lenses have been in use since the 19^th^ century [15–22]. The Stokes lens consists of two cylindrical lenses of equal power but opposite sign, arranged to rotate relative to each other. This configuration enables continuous modulation of astigmatism: from zero astigmatism when the axes of the cylindrical lenses are aligned, to twice the cylinder power when their axes are crossed perpendicularly. Originally introduced by George Stokes for astigmatism measurement in the eye, the lens gained broader adoption through the fixed version later proposed by Edward Jackson, now widely known as the Jackson Cross-cylinder [23]. Recently, Albero-Moreno et al. [7,24] characterized and employed three Stoke lenses adapted to a conventional phoropter in order to determine astigmatic correction. These technologies not only align with the power vector framework for refraction, but also offer a level of versatility and operational efficiency that conventional methods lack.

Beyond their technical advantages, the simplicity and ease of manipulation of TLLs also introduce exciting new possibilities in subjective refraction. Their user-friendly design makes it feasible for individuals without prior optometric training to handle and adjust the lenses under the guidance of a professional. This opens the door to innovative, patient-centered approaches, including a “do-it-yourself” (DIY) approach in which the professional provides instructions while the patient actively participates in refining their refractive correction. Such methods have the potential to empower patients to play an active role in the process.

In light of these developments, this study aims to evaluate the precision and accuracy of a novel DIY subjective refraction approach using a TLL and a Stoke lens. By comparing this approach to traditional (TR) professional-guided methods, we seek to explore its feasibility and clinical potential while addressing its ability to maintain high standards of refractive accuracy.

## Methods

### Participants

A total of 66 subjects aged between 18–58 years were included in the present study. Inclusion criteria were no history of known ocular disease, no previous ocular surgery, spherical error under ±15 D and astigmatism under ±3.5 D. The refractive error ranges have been selected according to the ranges offered by the devices employed as explained below.

The study was approved by the Swedish Ethical Review Authority, and the study procedures adhered to the tenets of the Declaration of Helsinki. The data collection began on 3rd October 2024 and ended on 6th December 2024. Each participant signed an informed consent form after receiving a detailed explanation of the purpose, nature, and possible consequences of the study.

### Measurement device

A manual TLL [25] and a Stokes lens [26], aligned with each other, were placed in front of the subject’s eye. The compensation ranges of these devices are ± 15 D in spherical error, provided by the TLL, and ±3.5 D in cylinder compensation, provided by the Stokes lens which includes two cylinder lenses of ±1.75 D in cylinder power. Both TLL and Stokes lens have been previously characterized for these ranges and have shown a sensitivity (minimum increment) of ±0.13 D and ±0.24 D in spherical and cylinder power variation, respectively.

To ensure the correct positioning of the participants and their comfort during the measurements, a chin rest and a head rest were used. The contralateral eye was occluded during the process, and participants were instructed to use the hand they felt most comfortable with to rotate and adjust the lenses. Illumination was checked with a light meter close to subject’s eye and the same illumination level and room temperature were maintained for all measurements to prevent changes in environmental conditions among participants.

In the DIY refraction method, the participant actively participated in adjusting the optical elements of the refraction system, specifically the manual TLL [25] and the Stokes lens [22], under the optometrist’s guidance. This method was designed to identify the refractive error of subjects that provided optimal VA while maintaining a structured and reliable process. Three consecutive measurements were taken under repeatability conditions [27,28]. The subjective refraction values, the achieved VA, and the time needed to conduct the whole refraction process were recorded. A traditional subjective refraction was also carried out once following the standard procedure of Maximum Plus to Maximum Visual Acuity, and cylinder and axis were refined with ±0.25 D Jackson Cross-cylinders with a commercial phoropter. The optotype, an Early Treatment of Diabetic Retinopathy Study (ETDRS) chart in logMAR notation (Topcon CC-100), was showing lines of five randomly ordered logMAR letters. Refractive error, final VA, and time of measurement were recorded.

### DIY refractive examination procedure

#### 1. Subject instruction and training:

Before the procedure, patients were given a detailed explanation of the process, including the purpose of the liquid and Stokes lenses and their role in achieving clear vision. Instructions emphasized the need to rotate the lens dials very slowly and to focus on recognizing the letters displayed on the optotype screen instead of focusing only on sharpness. Demonstrations were provided if necessary, ensuring the subject understood their role and they felt comfortable with the procedure.

#### 2. Spherical equivalent adjustment:

This step consisted of leading the circle of least confusion (CLC) to retina, achieving the spherical equivalent value. The process began with the TLL preset to a slightly blurred starting point by adding +1.00 D of spherical plus power to the objective refraction results, achieved with a Tonoref III autorefractometer, in order to control accommodation. The participant was instructed to rotate the liquid lens dial slowly reducing the positive power (counterclockwise rotation from the subject’s side), adjusting the spherical power while observing the optotype. They were asked to stop adjusting as soon as the letters appeared recognizable. At this point, they were asked to read the letters aloud as confirmation so the optometrist could proceed decreasing the size of the line of the optotype. This process was repeated until the subject did not show further improvements in recognizing the optotype. A duochrome test was performed as a final refinement step, aiming to achieve a balanced visual response between the red and green backgrounds. The optometrist recorded the spherical power setting corresponding to the M component.

#### 3. Astigmatic component adjustment (J_0_ and J_45_):

Due to the properties of the Stokes lens, it generates pure astigmatic power along a specific meridian, allowing astigmatic component values to be adjusted independently according to the axis orientation without changing the position of the CLC. The Stokes lens was oriented with its axes aligned at 0° and 90° for determining J_0_, and at 45° and 135° for determining J_45_. The subject was asked to rotate the lens until the image did not improve anymore. This process was repeated twice, one to achieve J_0_ and another to achieve J_45_, in random order.

#### 4. Visual acuity assessment:

The final combination of spherical and astigmatic corrections (M, J_0_, J_45_) was converted to polar notation (M, [J_α_ x α]) [8] and the cylindrical component in polar form was introduced into the Stokes lens by the optometrist for logMAR VA measurement.

### Statistical analysis

Data were measured from only one eye of each participant to avoid the possibility of artificially reducing the confidence interval around the limits of agreement (LoA) [29]. Descriptive statistics were used to summarize the basic demographics of the obtained results regarding refraction, VA, and required time. Data with a normal distribution were described as the mean with the standard deviation (SD), and data with non-normal distribution were described using the median and interquartile range (IQR). The normality of the distribution was evaluated with the Shapiro-Wilk test.

The following metrics were used to describe the repeatability of the DIY refraction approach: the within-subject standard deviation (${\text{S}}_{\text{W}}$) estimated from the one-way analysis of variance (ANOVA) with the subject as a factor; and the coefficient of repeatability (CoR), which was calculated as $\text{1}.\text{96}\times {\text{S}}_{\text{W}}$ [29].

The agreement between the traditional method with the DIY refraction was evaluated by the Bland-Altman analysis for refractive components, VA, and time [30]. To determine whether the differences between methods were statistically significant or not, paired Student t-test was used when distribution followed normality, and the Wilcoxon signed-rank test was used in non-normal distributions. The threshold for statistical significance was set at a p-value < 0.05.

## Results

**Fig 1:**
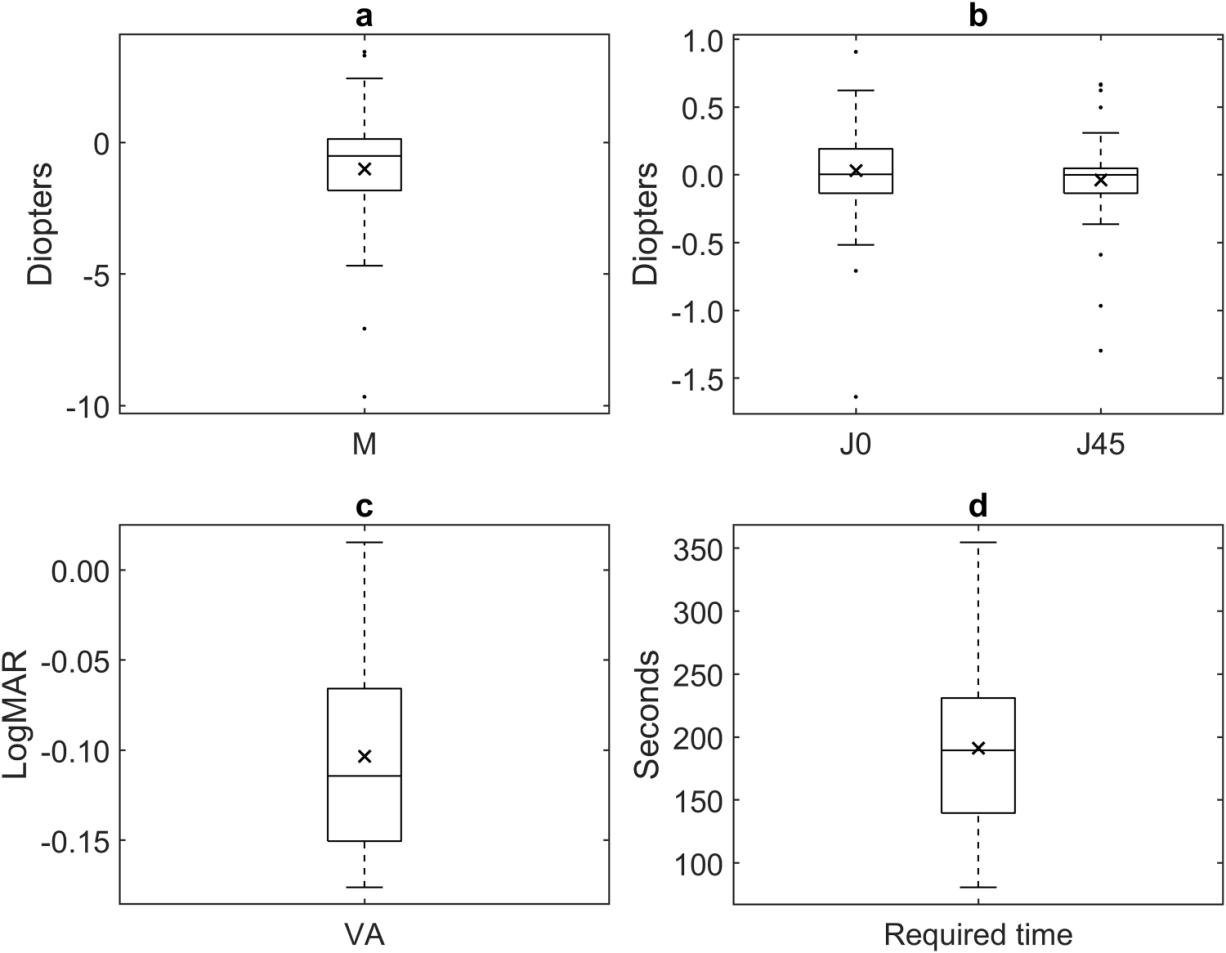
Boxplot of the average values of the three measurements obtained for each variable.

Although sixty-nine subjects were initially recruited for the study, three of them were excluded because they did not meet the inclusion criteria. One subject had myopia greater than −15 D, one had astigmatism greater than −3.5 D, and one had incomplete measurements. Then, a sample of 66 subjects was included in the study. The mean age of the sample was 29 ± 9 years with an average sphero-equivalent of −0.98 ± 2.28 D (sphere: −0.68 ± 2.33 D [from −10 to 5.5 D], cylinder: −0.59 ± 0.69 D [from 0 to −3.5 D]) and 48% were female. Fig 1 illustrates the distribution of the mean values from the three repetitions for the DIY subjective refraction components M, J_0_, and J_45_, the achieved VA, and the required time.

### Repeatability of the DIY refraction method

**Fig 2:**
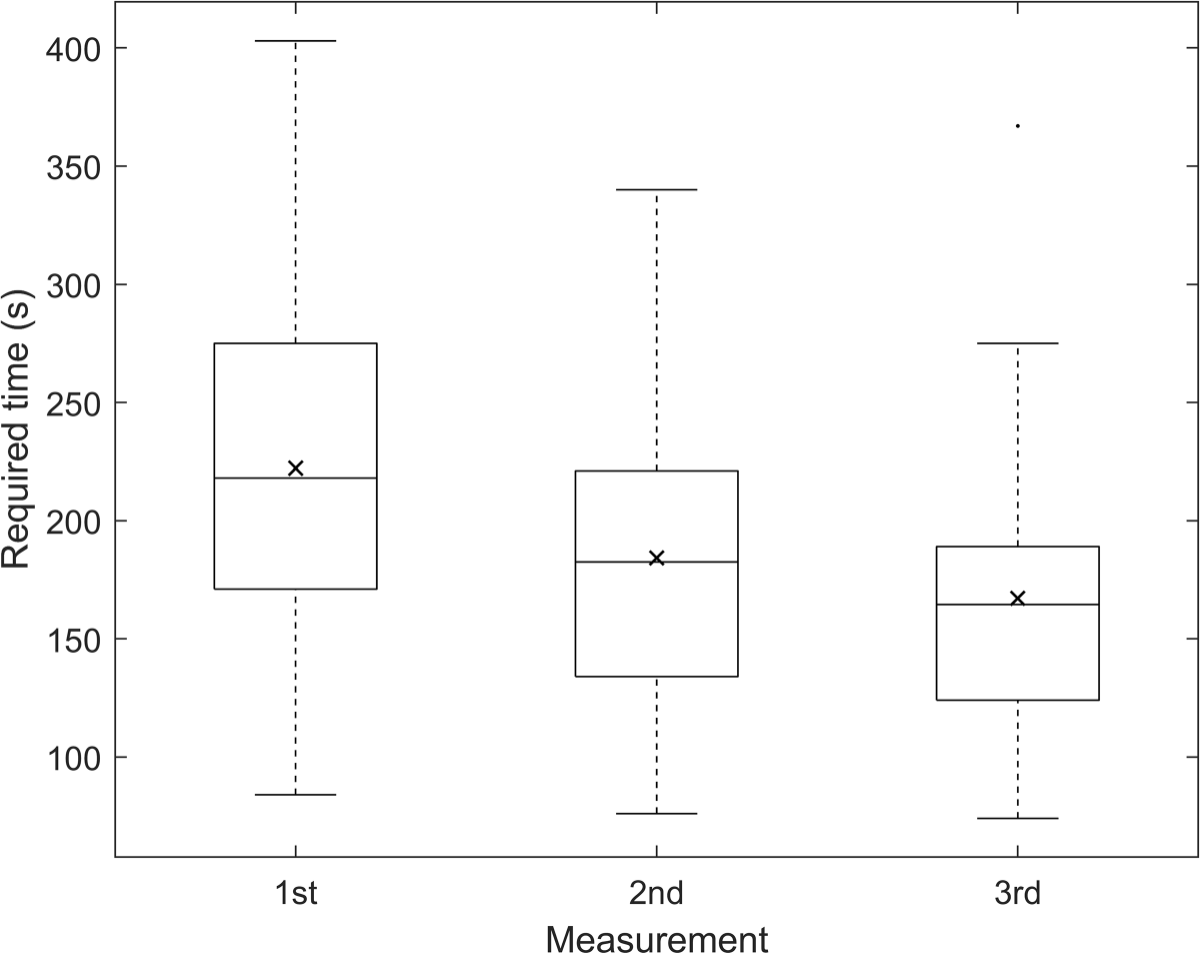
Boxplot showing the required time (in seconds) for performing the measurements across three consecutive attempts.

The mean of the within-subject range for three repeated measures for M was 0.26 ± 0.21 D, while for J_0_ and J_45_ were 0.16 ± 0.12 D and 0.14 ± 0.14 D, respectively. The CoR for M component was ± 0.38 D, whereas CoRs for the astigmatic components were ±0.21 D for J_0_ and ±0.21 D for J_45_. Concerning VA, the mean of the within-subject range for VA between measurements was 0.04 ± 0.04 logMAR. The CoR for VA was ± 0.06 logMAR. Regarding the required time for the measurement, the mean of the within-subject range between measurements was 74 ± 45 s, while the CoR was ± 55 s. No statistically significant differences were found between repetitions in all parameters according to the one-way rANOVA (p-value(M) = 0.78, p-value(J_0_) = 0.47, p-value(J_45_) = 0.30, p-value(VA) = 0.19, p-value(time) = 0.81). Nevertheless, in the case of the required time for the measurement, its value reduces with practice (as can be seen in Fig 2, in which the boxplots for each of the three consecutive DIY measurements are shown).

### Differences between TR refraction and DIY refraction methods

**Fig 3:**
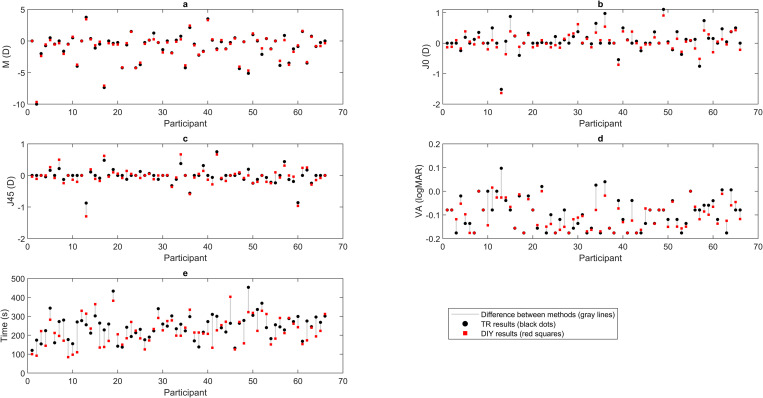
Scattering plot of individual measurements for each subject from the traditional method (black dots) and the do-it-yourself method (red squares).

The values obtained for each variable and each patient using the TR method and the DIY method are shown in Fig 3. The scatter plots display the results for each participant, with black dots representing TR values, red dots showing DIY values, and grey lines highlighting the differences between the two methods. For M, the values ranged from −10.00 to 3.75 D with the TR method and from −9.67 to 3.44 D with the DIY method. The maximum and minimum differences found in the M component are 0.99 and 0 D, respectively, where 92% of the cases show less than 0.5 D difference, and 52% are less than 0.25 D. For J_0_, the ranges were −1.52 to 1.11 D (TR method) and −1.64 to 0.91 D (DIY method). Similarly, for J_45_, the values ranged from −0.88 to 0.75 D (TR method) and from −1.30 to 0.67 D (DIY method). The maximum differences found for the astigmatic components are 0.49 and 0.42 D for J_0_ and J_45_, respectively, while the minimum for both is 0 D. Additionally, 55% of the J_0_ differences and 70% of the J_45_ differences are under the value of 0.125 D. Visual acuity ranged from −0.18 to 0.10 logMAR with the TR method and from −0.18 to 0.02 logMAR with the DIY method. Finally, measurement time ranged from 120 to 453 seconds for the TR method and from 84 to 403 seconds for the DIY method.

### Agreement between methods: Spherical component

**Fig 4:**
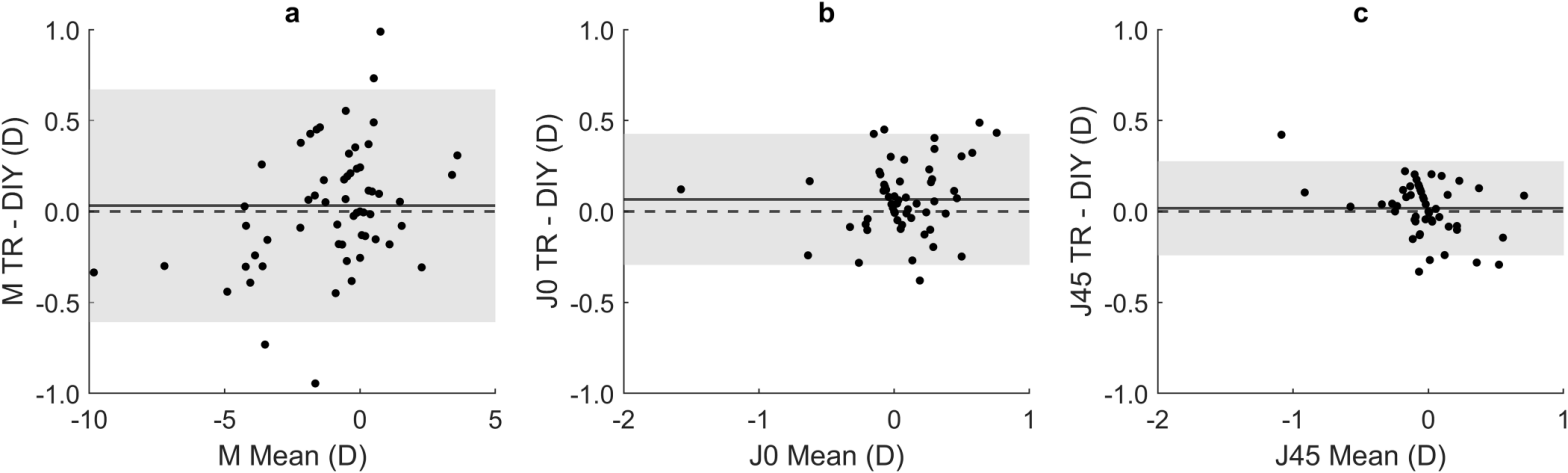
Bland-Altman plot showing the agreement between the traditional method and the do-it-yourself method for the refractive components. Continuous line represents the mean, dashed line represents the 0 D value and shadow grey area represents the 95% limits of agreement.

Fig 4a shows the Bland-Altman plot for M component between TR and DIY with the mean difference line and the 95% LoA. Given that the M component was measured only once for the TR method but three times for the DIY, the mean values between the three DIY measurements were used in the comparison. Mean difference was close to zero (0.03 D) whereas the 95% LoA ranged between ±0.64 D. Difference did not show statistical significance according to paired t-test (p = 0.386).

### Agreement between methods: Astigmatic components

Fig 4b and Fig 4c show the Bland-Altman plots between the TR method and the DIY method for both astigmatic components (J_0_ and J_45_). The mean values of the three repetitions for J_0_ and J_45_ for the DIY method were used. In both cases, mean differences values were close to 0 D, but J_0_ presented slightly higher values obtained with the TR method. The agreement of J_0_ showed a 95% LoA of ±0.36 D whereas J_45_ was ± 0.26 D. Differences were statistically significant for J_0_ (p = 0.004) but not for J_45_ (p = 0.29) according to the paired t-test.

### Agreement between VA

The VA data did not conform to the normal distribution (p < 0.003, Shapiro–Wilk). Consequently, non-parametric statistical methods were employed to assess differences among methods. The Wilcoxon signed-rank test did not reveal significant differences in VA between TR and DIY refraction (p = 0.202). The median of the difference for VA was 0.00 logMAR, and the interquartile range was 0.04 logMAR (from −0.03 to 0.01 logMAR). In approximately 53% cases the VA was equal or better with DIY refraction in comparison with TR refraction. There was only one case where the differences between VA for both refractions exceeded one logMAR line.

### Agreement between the required time for measurement

**Fig 5:**
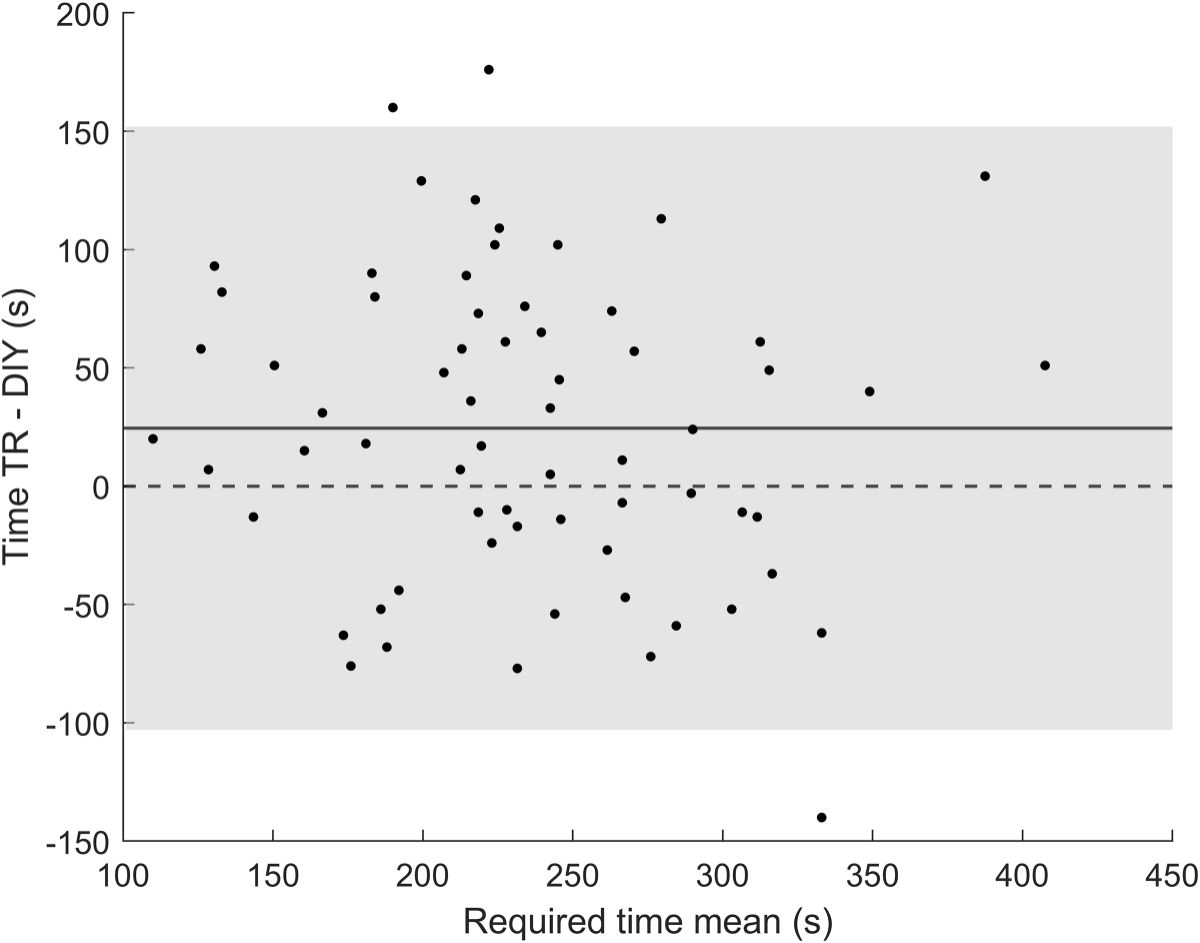
Bland-Altman plot showing the agreement between the traditional method and the do-it-yourself method for the time required. Continuous line represents the mean, dashed line represents the 0 D value and shadow grey area represents the 95% limits of agreement.

Given that the required time demonstrated a strong learning curve (as already shown in Fig 2), only data from the first measurement was used to assess the agreement between the TR and the DIY method. This approach ensured that participants were considered novices for both methods, minimizing potential bias. Under these conditions, the mean required time for the DIY method was 222 ± 74 s while the mean required time for the TR method was 246 ± 66 s. Fig 5 shows the Bland-Altman plot comparing the TR subjective refraction with the DIY method. The mean difference was approximately 24 s, with LoA of ±127 s, the DIY method was faster in 62% of the sample, with 30% of participants completing the task at least 60 seconds faster. These differences between methods were statistically significant, as determined by the paired t-test (p = 0.003).

## Discussion

In this study, a novel method for self-assessing non-cycloplegic subjective refraction was developed and evaluated, with the aim of minimizing clinician involvement. This new method utilizes as measuring tools only a manual TLL and a Stokes lens. The repeatability across three measurements, as well as the agreement between traditional subjective refraction using a commercial phoropter and this new method, were investigated.

### Repeatability analysis

For refractive components, the highest coefficient of repeatability was observed for the M component, being ±0.38 D, while the astigmatic components (J_0_ and J_45_) showed similar results to each other, being ±0.21 D. This is not surprising, as the M component relates to spherical refractive errors which are generally much higher than astigmatic errors. Other studies have reported ${\text{S}}_{\text{W}}$ values ranging from ±0.13 D to ±0.32 D for subjective refraction processes conducted by eye care professionals [4,6,31–36]. Nevertheless, several investigations characterized new self-refraction techniques, but no data about repeatability was provided. According to Rosenfield and Chiu [37] a subjective refraction method can be considered clinically repeatable when the 95% LoA for the sphero-equivalent (M), calculated as $\text{1}.\text{96}\times {\text{S}}_{\text{W}}$ (CoR), are up to ±0.50 D. In this study, the repeatability for the M component was within this limit.

Unlike the sphero-equivalent component, there is no clinical repeatability threshold well-established in the literature for the astigmatic component. Therefore, we can only compare the obtained values with previous studies. For example, Raasch et al. [36] studied the repeatability of subjective refraction in myopic and keratoconic subjects. In forty myopic subjects, they found a CoR of ±0.23 D for J_0_ and ±0.16 D for J_45_. Leinonen et al. [38] found CoR values ranging from ±0.57 D (vertical component) to ±0.71 D (horizontal component) in 99 subjects with good VA. More recently, Huang et al. [35] reported a CoR of ±0.59 D for J_0_ and ±1.17 D for J_45_ in 65 subjects fusing a tele-controlled subjective refraction.

In terms of VA, the coefficient of repeatability in our study is ± 3 logMAR letters (±0.06 logMAR) [39]. Traditionally, studies using test-retest SDs (${\text{S}}_{\text{W}}$) of VA have found repeatability to be within two to three letters (0.04 to 0.06 logMAR) [39–44]. Therefore, the repeatability of VA for this new method was within the traditionally reported limits in previous studies.

The required time showed a CoR of ±55 s and could be defined as statistically repeatable. However, there was a strong learning effect between measurements (as was shown in Fig 2). There was a decrease in the median and mean time, and in general in the whole distribution of values (the height of the boxes, the interquartile range, and the length of the whiskers decreased progressively), suggesting, as expected, that participants performed the tasks faster with practice.

### Agreement analysis

The distribution of differences in spherical equivalent power between the DIY and conventional refraction methods was centered around zero. This indicates that there is no systematic tendency toward over-minus or over-plus correction. Thus, the DIY approach provides spherical corrections that are comparable to those obtained through conventional subjective refraction.

The 95% LoA for the M component in this study were not less than ±0.5 D (Fig 4), which are the current limits of clinical significance for subjective refraction [37]. Many other studies have also developed new subjective refraction methods to find an alternative to the traditional subjective refraction but to reach the current threshold [3,5–7,33,35,45–57] Specifically, some of them focused on self-refraction techniques as we did in this study. Leube et al. [53] used a pair of Alvarez lenses that could be rotated. Eighteen patients were asked to self-adjust the power by moving the pair of lenses in three meridians (0°, 60°, and 120°, respectively). They reported LoA when comparing it with the traditional subjective refraction of ±1.10 D for M, and ±0.78 D and ±0.62 D for J_0_ and J_45_, respectively. Wisse et al. [45] evaluated the results of web-based refraction (www.easee.online, Easee, Amsterdam, The Netherlands) compared to TR. They found the LoA to be ± 1.35 D for M in 121 eyes of 64 subjects. Tousignant et al. [46] evaluated the efficiency of a self-test auto-refractor (eyenetra.com, EyeNetra, Boston, MA, USA) when patients perform the measurement themselves. They compared the results of TR and unaided self-refraction in 36 patients and found a LoA of ±1.40 D for the M component. They also evaluated the differences between the methods in terms of cylinder and axis. No LoA was reported, but they stated that no statistically significant differences were found between the two methods for cylinder and axis separately (Wilcoxon sign test, p > 0.05). Annadanam et al. [57] used a commercial model USee (USee, Global Vision 2020, Easton, MD, USA), which consists of adjustable lenses in an eye frame, in sixty-seven subjects. When compared to TR, they obtained two LoA: one for the right eye of ±1.43 D and another for the left eye of ±1.72 D. Ilechie et al. [56] also compared a self-refraction method with adjustable spectacles to TR. Furthermore, they obtained their results under cycloplegia. They found a LoA of ±0.77 D. As can be seen, none of the self-refraction methods meet the criteria of ±0.5 D for the 95% LoA. Nevertheless, the DIY approach introduced in this study shows the closest results to this threshold.

Regarding the astigmatic components, we obtained a mean difference between methods close to zero for both components. At the statistical level, significant differences were found for J_0_, but not for J_45_. Since statistical significance does not always correlate with clinical relevance, it would be useful to establish a clinical threshold for astigmatic components, as has been done in the literature for the sphero-equivalent. Regarding the LoA, we obtained values for J_0_ and J_45_ equal to ±0.36 D and ±0.26 D, respectively (Fig 4). Other studies have also described these LoA. For example, Leube et al. [53] reported LoA of ±0.78 D and ±0.62 D for J_0_ and J_45_, respectively. In addition, Albero-Moreno et al. [7] found a LoA of approximately ±0.25 D for J_0_ and a slightly superior LoA for J_45_ in a comparison between TR and a new subjective refraction method performed by an eye care professional using Stokes lenses. Therefore, it can be said that the LoA between TR and DIY for the astigmatic components are at least within previously reported limits for new subjective refraction methods, or better than those.

No significant differences were found for VA between TR and DIY. The median of VA was the same and the interquartile range was 0.04 logMAR, which is a difference of two letters between one method and the other. A relevant fact was that only one participant reported differences of more than one line of VA. Therefore, it could be considered that this difference is not clinically relevant since other factors such as subject fatigue or tear film conditions could influence the measurements [58]. Future investigations could compare the final VA in a double-blind manner, where neither the participants nor the professionals know which refraction is being used.

The required time showed large LoA, up to ±2.12 min. This shows a high variability and dependence of the subject’s response time. Still, as can be seen in Fig 5, the mean difference between TR and DIY was set at 24 s, which would imply a reduction in time when comparing DIY with respect to TR. As mentioned above, we only used the first DIY measurement to make the comparison, so neither method had an advantage over the other in terms of familiarity. Additionally, the shorter times seen in the DIY condition may be attributed to multiple factors. People might feel more in control when they adjust the system themselves, which could help them make decisions faster. It is also possible that the DIY method would be more intuitive, as it eliminates the need for verbal communication and external interpretation, which might reduce delays. Moreover, the experimenter in the TR method might need to pause to think about the subject’s feedback and make changes. Finally, people might act differently depending on the situation. For example, when people think they are in charge of a DIY project, they might pay more attention and be more focused, which could also make the project take less time.

We have talked about the learning process present in the DIY method and we cannot exclude that a similar factor affects the traditional subjective refraction. This would be an interesting aspect to study in future work. Regarding the time required for refraction, Rodríguez-Lopez et al. [54] did a conscientious job in summarizing the time required for other different subjective refraction methods. Considering binocular refraction, the time required ranges from 1.56 min to 22 min, with an approximate mean of 6.25 ± 2.00 min [4,45,48,50,55,59]. Of all these studies, only one was self-refraction, and that was the method characterized by Wisse et al. [45] with a required time of 22 ± 10 min. The mean time required for the DIY method was 3.7 ± 1.2 min, shorter than for TR, although new methods of subjective refraction can achieve faster results.

However, this DIY approach has some limitations. One of the most important factors that may affect performance is the level of understanding that participants achieve in the initial explanation. They were asked to stop rotating the lens as soon as they could recognize the letters on the screen, even if they were slightly blurred. This was an attempt to avoid overstimulating accommodation and adding more negative values than necessary. Another factor is related to the Stokes lens and the amount of astigmatism. Participants with low astigmatism tended to be more hesitant in their responses, because the change caused by the Stokes lens was initially challenging for the participants to appreciate until they realized a deterioration in the visibility of the optotype letters compared to the prior step. High astigmats were more confident in this regard. In addition, since both lenses are tunable, any misalignment between the subject’s eye and the lenses could affect the measurements and the final VA. Moreover, this is only a first attempt where the eye-care practitioner still plays an active role in the process. Further studies should aim to reduce the clinician’s involvement and refine the examination protocol in a larger sample of participants. This future work should also focus on how the lack of professional advice and knowledge can influence subjective refraction results. This is important not only in a young and healthy population without visual problems, such as amblyopia or accommodative disorders, but also in other populations of different ages, with ocular pathologies, or with physical or mental disabilities.

In conclusion, the DIY method provides repeatable measurements and shows a high learning effect in terms of time required. Although it cannot be regarded as a replacement for traditional subjective refraction, and it is a first attempt and not yet fully automatic. This new method achieves results comparable to those of previously reported self-refraction methods and could be considered as a convenient option in certain situations such as where there is a shortage of eye care professionals, or where the implementation is deemed to be straightforward, for example in developing countries. For this to become a reality, further research should be conducted to evaluate the DIY method outcomes without minimal supervision and in larger population samples.

## Supporting information

S1 Data Inclusivity in global research questionnaire. (DOCX)
